# Performing a vibrotactile discrimination task modulates finger representations in primary somatosensory cortex

**DOI:** 10.1152/jn.00428.2022

**Published:** 2023-09-06

**Authors:** Finn Rabe, Sanne Kikkert, Nicole Wenderoth

**Affiliations:** ^1^Neural Control of Movement Laboratory, Department of Health Sciences and Technology, ETH Zürich, Zurich, Switzerland; ^2^Neuroscience Center Zurich (ZNZ), ETH Zurich and University of Zurich, Zurich, Switzerland; ^3^Spinal Cord Injury Center, Balgrist University Hospital, University of Zürich, Zurich, Switzerland; ^4^Future Health Technologies, Singapore-ETH Centre, Campus for Research Excellence and Technological Enterprise (CREATE), Singapore

**Keywords:** primary somatosensory cortex, somatosensation, somatotopy, vibrotactile, working memory

## Abstract

It is well established that vibrotactile stimuli are represented in somatotopic maps. However, less is known about whether these somatotopic representations are modulated by task demands and maybe even in the absence of tactile input. Here, we used a vibrotactile discrimination task as a tool to investigate these questions in further detail. Participants were required to actively perceive and process tactile stimuli in comparison to a no-task control condition where identical stimuli were passively perceived (no-memory condition). Importantly, both vibrotactile stimuli were either applied to the right index or little finger, allowing us to investigate whether cognitive task demands shape finger representations in primary somatosensory cortex (S1). Using multivoxel pattern analysis and representational similarity analysis, we found that S1 finger representations were more distinct during the memory than the no-memory condition. Interestingly, this effect was not only observed while tactile stimuli were presented but also during the delay period (i.e., in the absence of tactile stimulation). Our findings imply that when individuals are required to focus on tactile stimuli, retain them in their memory, and engage in active processing of distinctive stimulus features, this exerts a modulatory effect on the finger representations present in S1.

**NEW & NOTEWORTHY** Using multivoxel pattern analysis, we found that discrimination task demands shape finger representations in the contralateral primary somatosensory cortex (S1), and that somatotopic representations are modulated by task demands not only during tactile stimulation but also to a certain extent in the absence of tactile input.

## Graphical abstract

**Figure:**
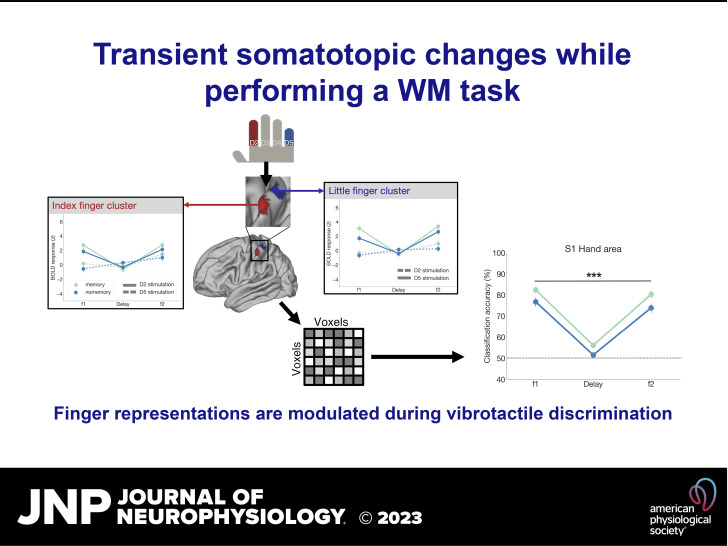

## INTRODUCTION

Topographic representations such as the somatotopic map in the primary somatosensory cortex have been shown to be ubiquitous in the cerebral cortex of mammals. They consist of orderly representations of receptor surfaces on different body parts (1–4). These somatotopic maps are specific to the point where the sensation of each finger can be assigned to its own cortical region, so-called finger representations (5–9).

Importantly, finger-specific somatosensory representations can be activated by tactile or proprioceptive stimulation. However, they can also be modulated through other mechanisms such as *1*) attempted movements that do not produce overt motor output or the associated tactile or proprioceptive feedback (10–12), *2*) observed (13) or actively imagined touch (14), *3*) or directing attention to a specific finger (15, 16).

Here, we ask whether somatotopic representations in the primary somatosensory cortex (S1) are modulated by task demands underlying vibrotactile discrimination, that is, when somatosensory stimuli are *1*) encoded, *2*) kept in memory, and *3*) compared with a second stimulus for subsequent decision making (17). Previous research suggests that S1 is not only involved in vibrotactile stimulus perception but that it might contain task-relevant information also during the working memory (WM) delay period of the task, that is, in the absence of acute tactile stimulation.

Single-unit recordings in nonhuman primates revealed neural activity in the S1 hand area while subjects had to match an object with a specific surface to a previously presented surface stimulus (18, 19). The authors observed cells that were not only active while the tactile stimulus was present but also sustained their firing during the WM delay period. This suggests that primary sensory cortices could serve as a memory buffer for stimulus information (20), an idea that has been conceptualized as the “sensory recruitment” model of WM (21, 22). According to this model, WM is maintained in those brain regions that are involved in encoding sensory stimuli. However, it is important to note that other nonhuman primate studies do not suggest the involvement of S1 during the WM delay period (23). In addition, a human neuroimaging study could show that the average activity level of S1 (as detected by a “mass-univariate” statistical approach) is not significantly larger during the WM delay period than during a control condition (24).

Other studies, by contrast, have used multivoxel pattern analysis (MVPA) that can detect stimulus information in spatially distributed patterns of activation in a region of interest (ROI) (25). When exposed to a stimulus, stimulus-selective neurons are activated that can be quantified using tuning curves. The product of multiple tuning curves can be defined as the neural population code (26, 27). A linear decoder applied to functional magnetic resonance imaging (fMRI) data of a group of single voxels has the ability to estimate which information is kept in the neural population code (28) and how this representation might change due to task demands. Neuroimaging studies utilizing MVPA found that features like vibratory frequencies were represented during the delay period, mainly in associative brain regions, that is, posterior parietal and frontal regions (29, 30). Interestingly, features of spatial layout stimuli could be decoded from S1 during the delay period (31), even though such stimulus representations might fade out and in over time, especially when a temporal structure of the delay period can be anticipated (32). Similar to the aforementioned observations in nonhuman primates, spatial layouts could be decoded during the delay period from the S1 area that usually represents the hand (31). This area has been characterized by its fine-grained finger representations (5–9). Even though it is well-known that stimulating different fingers evokes spatially distinct activity in S1 in accordance with its somatotopic map, we were interested in quantifying whether these finger-specific representations are additionally modulated by cognitive task demands. To answer this question, we collected fMRI data while participants either *1*) discriminated between two tactile stimuli that were both presented either to the index or little finger (i.e., requiring the participants to keep the first stimulus in memory and compare it to the second), or *2*) were exposed to identical tactile stimuli without actively processing them. Brain activity during stimulus presentation but also during the delay period (i.e., when no tactile input was provided) was analyzed with multivariate techniques to estimate whether index versus little finger representations change depending on task demands. We hypothesized that high classification accuracies and distinct neural representations of stimulating the index versus little finger align with the idea of high finger specificity and narrow tuning curves in the S1 neuronal population (33). Low classification accuracies and dissimilarities between fingers, by contrast, indicate that S1 activity is less finger specific suggesting that tuning curves are broader. Although it is highly likely that task demands modulate somatosensory representations during the tactile stimulation periods, we additionally explored whether S1 representations would also differ during the delay period, that is, in the absence of tactile stimuli.

## MATERIAL AND METHODS

### Participants

Thirty young healthy volunteers (19 females; mean age = 24.48, SE = 0.44) participated in our study. Our sample size was comparable with those in previous reports on fMRI decoding of WM content using discrimination tasks (29, 34). All participants were neurologically intact and reported to be right handed. All of them gave written informed consent and the study protocol was approved by the local ethics committee (BASEC-Nr. 2018-01078). Three participants had to be excluded due to excessive head motion based on our criterion (see *Preprocessing of fMRI Data* for more detail).

### Experimental Procedure and Tasks

#### Tactile stimuli.

Vibrotactile stimuli (duration = 2 s, sampling rate = 1 kHz) were applied to the right index or right little finger using an MR-compatible piezoelectric device (PTS-T1, Dancer Design, UK). We selected these fingers as they have the largest inter-finger somatotopic distance (8, 9, 35, 36), allowing us to robustly detect the modulation of somatotopic representations by the vibrotactile discrimination task. The one-bin piezoelectric wafers were mounted to the fingertips using custom 3-D-printed retainers that were fixed with a Velcro strap. Participants were asked to report any tingling sensation in case the retainer was mounted too tightly. The stimulation consisted of mechanical sinusoids that were transmitted from the testing computer to the piezoelectric device using a C Series Voltage Output Module (National Instruments) and the in-house NI-DAQmx driver.

#### Sensory detection threshold estimation.

To ensure similar task difficulty across runs of the main experiment (37), we determined the sensory detection threshold (SDT) for both fingers before starting the main experiment. SDT was defined as the stimulation intensity at which the participants detected the stimulus 50% of the time. We stimulated each finger only once per trial at base frequency (20 Hz) and participants were asked to press a button upon detection of a stimulation. To reliably estimate SDT, we applied a conventional Bayesian-based Quest procedure (QuestHandler in PsychoPy). After each detected or undetected stimulus, the algorithm searched for the most probable psychometric function via maximum likelihood estimation over the course of 25 trials starting with a stimulation amplitude of 0.1 V (38). The Weibull psychometric function was calculated using the following formula:

(*1*) Ψ(x)=δγ+(1−δ)[1−(1−γ)exp(−10β(x−T+∈))],

where *x* is the stimulus intensity in volts and *T* is the estimated sensory detection threshold. This procedure was performed before the first run and each stimulus intensity was multiplied by a factor of 2 to ensure suprathreshold performance (similar to that detailed in Ref. 39). If the percentage of correctly discriminated memory trials in a run was below 60% or above 90%, then we redetermined the SDT using a shortened version of the Quest procedure. In such a case, we started the Quest procedure with the previously determined stimulation intensity to reduce the number of iterations (new iterations = 7 trials). This procedure was applied to keep task difficulty at comparable levels throughout the experiment (Supplemental Table S5, https://doi.org/10.6084/m9.figshare.24049257).

We analyzed changes in behavioral performance that occurred across runs, potentially due to perceptual learning, cooling of the fingertips, or fatiguing effects using a repeated-measures two-way ANOVA (2 fingers × 4 runs; Fig. 2*B*).

#### Main experimental task.

**Figure 1.**
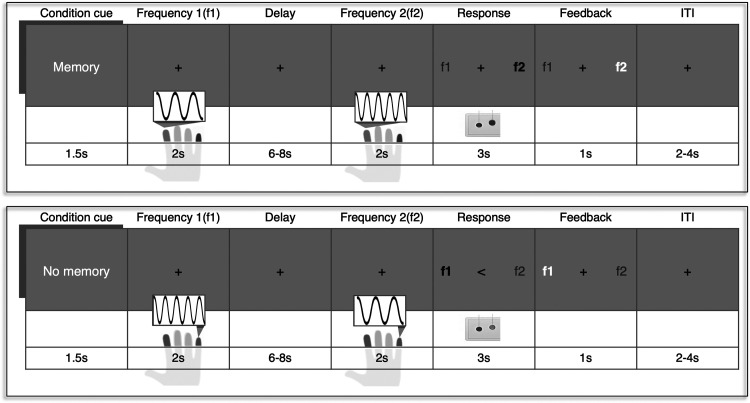
Vibrotactile discrimination task. During memory trials (*top*), two vibrotactile stimuli that differed in frequency were consecutively applied to the same finger (in this example the index finger). Both stimulations were separated by a jittered delay period, during which participants had to keep the first stimulation frequency (f1) in memory to compare it to the frequency of the second stimulus (f2). During the 3-s response period, participants had to indicate which of the two stimulation frequencies (f1 and f2) was higher by means of a left-hand button press. The mapping between the discrimination response and which button to press was indicated on the screen and randomized across trials. Subjects received feedback on whether their response was correct or incorrect. The target finger (index or little finger) was intermixed within a run and the intertrial interval (ITI) was jittered between 2 and 4 s. During no-memory trials (*bottom*), vibrotactile stimulations and visual information remained the same. However, participants were instructed not to focus on the stimulation and also not to compare the vibrotactile stimuli. They simply had to press the button indicated by the arrow in the middle of the screen.

The main experimental task was generated using PsychoPy (40). The experimental task consisted of memory and no-memory trials, each binned into four consecutive trials. During a memory trial, participants performed a two-alternative forced choice (2AFC) discrimination task. Two vibrotactile stimuli were consecutively applied to the same finger (i.e., the index or the little finger), separated by a jittered 6–8 s delay. We targeted cutaneous mechanoreceptors that respond to stimulations in the flutter range (41). One of two stimuli vibrated at 20 Hz (2-s duration at SDT intensity) whereas the vibration frequency of the other stimulus varied between 22, 24, or 26 Hz (same duration and intensity). Participants had to indicate by means of a button press whether the first or the second stimulation was higher in frequency (half of the participants) or whether the first or the second stimulation was lower in frequency (the other half of the participants), following previously published procedures (39, 42, 43). Responses were recorded via index and middle finger button presses of the other (left) hand using an MR-compatible fiber optic device. We randomized the order of how the response options (f1 and f2) appeared on the screen on a trial-by-trail basis to prevent somatotopy-specific anticipatory motor activity. After a 3-s response period, participants received visual feedback (1 s) indicating whether their response was correct (highlighted by green color) or incorrect (red; Fig. 1). Participants were instructed to focus their gaze on the fixation cross in the middle of the screen during the complete trial. Vibrotactile stimuli trials targeted either the index or the little finger and which finger would be stimulated per trial was counterbalanced across each run.

To disentangle cognitive processes required for solving the vibrotactile discrimination task from general responses to the stimuli, we also included no-memory trials. During a no-memory trial, participants received the exact same vibratory stimulations as during memory trials, but they were instructed not to focus on the stimuli or on their vibration frequencies. During the response period, subjects were informed by a visual cue (pointing arrow) which button to press. To ensure participants did not switch cognitive strategies, the indicated response was always contrary to the response that would be expected when correctly discriminating both frequencies. If participants reported they switched their cognitive strategies during the no-memory trials, we excluded them from any further analysis. Memory and no-memory trial conditions were separated into mini blocks of four trials. Participants were informed whether they had to perform the memory or no-memory task by means of a visual cue (1.5 s) at the beginning of each trial. Before the experiment, participants were familiarized with the memory and no-memory tasks by completing 12 trials.

The order of stimulus sites (stimulated finger) was counterbalanced both within and across mini blocks. Nonetheless, participants could predict the location of f2. However, this was the case for both the memory and the no-memory condition, and potential spatial attentional effects would equally occur in both. Stimulation frequencies were counterbalanced across the experiment. Each stimulus frequency was presented equally often in both memory and no-memory condition. Jittered timings for interstimulus interval (ISI, 6–8 s) and intertrial interval (ITI, 2–4 s) were randomly drawn from a uniform distribution. All participants completed four runs consisting of 48 trials each. Each run consisted of six memory and six no-memory mini blocks in a counterbalanced order.

### Behavioral Analysis

We defined the discrimination accuracy per participant as the percentage of correctly discriminated trials separately for each condition. We expected that greater frequency differences would facilitate discrimination between both tactile vibrations while the stimulus site should have no effect. We, therefore, investigated whether behavioral performances differed across frequency differences and across fingers using a two-way repeated-measures ANOVA.

### MRI Data Acquisition

Functional as well as structural MRI images were acquired on a Philips Ingenia 3 Tesla MRI (Best, The Netherlands) using a 32-element head coil. fMRI data were collected using an echo-planar-imaging (EPI) sequence acquiring 36 transversal slices centered at the bicommissural line and with whole brain coverage, though excluding most of cerebellum [repetition time (TR): 2 s, echo time (TE): 30 ms, spatial resolution: 3 mm^3^, field of view (FOV) = 222 × 222 mm^2^, 85° flip angle, slice orientation: transversal, SENSE factor (anterior-posterior, AP): 2, 472 functional volumes per run]. Anatomical images were acquired during SDT estimation using an MPRAGE T1-weighted sequence (TR = 7.7 ms, TE = 3.6 ms, FOV = 240 × 240 mm^2^, flip angle: 8°, resolution: 1 mm^3^, number of slices: 160, slice thickness: 2.2 mm, slice orientation: sagittal).

### Preprocessing of fMRI Data

Conventional preprocessing steps for fMRI data were applied to each individual run in native three-dimensional space, using FSL’s Expert Analysis Tool FEAT (v6.00; fsl.fmrib.ox.ac.uk/fsl/fslwiki). The following steps were included: motion correction using MCFLIRT (44), brain extraction using automated brain extraction tool BET (45), high-pass filtering (100 Hz), slice-time correction, and spatial smoothing using a 3-mm FWHM (full width at half maximum) Gaussian kernel using FEAT. Functional data were aligned to structural images initially using FLIRT (46), and optimized using boundary-based registration (47). Blood oxygen level-dependent (BOLD) EPI data were assessed for excessive motion using motion parameter estimates from MCFLIRT. If the functional data from a participant showed greater than 1.5 mm (half the voxel size) of absolute mean displacement, this participant was excluded from all further analyses.

To reduce physiological noise artifacts, these cerebrospinal fluid (CSF) and white matter were used to extract scan-wise time series which were then added to the model as nuisance regressors in addition to the standard motion parameters.

Structural images were transformed to Montreal Neurological Institute (MNI) standard space using nonlinear registration (FNIRT), and the resulting warp fields were applied to the functional statistical images.

### Definition of Regions of Interest

We used each individual participant’s T1-weighted image to create a cortical surface reconstruction by means of Freesurfer (48). We identified regions of interest (ROIs), specifically SI, anatomically for each subject based on the probabilistic Brodmann area parcellation provided by Freesurfer (49). More specifically, an S1 hand ROI was defined by combining Brodmann areas (BA) 1, 2, 3a, and 3b. We then converted this S1 ROI to volumetric space. Any holes were filled and nonzero voxels were mean dilated. Next, the axial slices spanning 2 cm medial/lateral to the hand area (50) were identified on the 2-mm MNI standard brain (min-max MNI *z*-coordinates = 40–62). This mask was nonlinearly transformed to each participant’s native structural space. Finally, we used this mask to restrict the S1 ROI and extracted an S1 hand area ROI. A similar ROI definition has been previously used (36, 51, 52). The S1 hand area ROI was used to both extract time-binned estimates as well as to decode information about the stimulus site during the delay period. All additional ROIs were created using standard ROIs from the Harvard-Oxford Cortical Atlas (i.e., IFG) and Juelich Histological Atlas [i.e., secondary somatosensory cortex (S2) and superior parietal lobule (SPL)] and were transformed into each participant’s native space.

### Univariate Analysis

First-level parameter estimates were computed per run using a voxel-based general linear model (GLM) based on the γ hemodynamic response function (similar as detailed in Ref. 24). Time series statistical analysis was carried out using FILM (FMRIB's Improved Linear Model) with local autocorrelation correction.

To find neural correlates of WM, we contrasted β estimates from the delay period during memory trials to those in no-memory trials. We then used a fixed effects higher-level analysis to average activity across runs for each individual participant. Finally, to make inferences on the population level, we computed a mixed-effects analysis (Flame 1). From this we obtained statistical group maps (*Z*-statistic images) for each contrast of interest, for example, contrasting memory delay activity to no-memory delay activity. *Z*-statistic images were thresholded using clusters determined by *Z* > 3.1 and statistical significance was determined at the cluster level [*P* < 0.05 family-wise-error-corrected (FWE)].

To further explore whether finger-specific activity levels were maintained in a somatotopic fashion, we first computed somatotopic ROIs by contrasting finger-specific activity during the first stimulation. We did this by contrasting activity associated with right index stimulations to right little finger stimulations, which elicited a finger-specific map (finger cluster) in the lateral part of S1 whereas the reverse contrast revealed more medially located activity (Fig. 3*A*). These S1 activity maps were in line with previous findings on finger somatotopy.

We then compared *z*-scored contrasts (index finger trials > rest or little finger trials > rest) between trials where either the index or the little finger was stimulated within each finger ROI. Finger ROIs activated during index and little finger stimulation *z*-statistic images were thresholded using clusters determined by *Z* > 3.1, *P* < 0.05 family-wise-error-corrected (FWE) cluster significance. Note that we identified participant-specific finger clusters based on local maxima closest to the group cluster peaks. Again, we computed a fixed-effects analysis as mentioned earlier. We then extracted *z*-stat values (e.g., index trials > rest within index finger cluster or little finger trials > rest) within the same cluster for each participant within their individual local maxima sphere (*r* = 8 mm) closest to the group finger peak during memory and no-memory trials at different timepoints (f1, delay, and f2).

Information retention in WM is not always reflected by constant delay activity, especially when the duration of the delay period can be somewhat anticipated (32). WM delay activity has been shown to decrease until shortly before memory retrieval when the remembered stimulus information is reactivated as suggested by an increase of neural oscillations in the θ band (32). We therefore hypothesized that the BOLD activity level would vary in a V-shaped fashion over the delay period. To test this hypothesis, we conducted a parametric modulation analysis. Our parametric modulation regressor was modeled to predict activity in three consecutive time-bins of the delay period in a V-shaped manner (Fig. 3*B*). The length of each time-bin equaled one TR (i.e., 2 s). Since we jittered the delay period between 6 and 8 s, we only modeled the first three time-bins of the delay period (2–6 s). The remaining time of the delay was modeled as a separate regressor of no interest. By contrasting memory and no-memory trials, we obtained *Z*-statistic images. These images were thresholded using clusters determined by *Z* > 3.1 and a familywise error-corrected cluster significance threshold of *P* < 0.05 was applied to the suprathreshold clusters.

To further visualize the results of the parametric modulation, we extracted activity estimates per time-bin. To do so, we modeled each time-bin of the delay period separately in a voxel-based general linear model (GLM) based on the γ hemodynamic response function. The remaining time of the delay was modeled as a separate regressor of no interest. We then extracted the *z*-scored estimates per time-bin within the previously defined S1 hand area ROI. All statistical maps were overlaid onto an MNI152 standard-space T1-weighted average structural template image (Supplemental Figs. S1 and S2, https://doi.org/10.6084/m9.figshare.23946972) and projected onto a cortical surface using Connectome’s Workbench (53).

### Variance Inflation Factor

To test whether multicollinearity between the parameter estimates in our GLM was sufficiently low, we calculated the variance inflation factor (VIF). This represents how much the variance of an individual regressor can be explained due to correlation to other regressors in our model (54). For each variable, VIF was computed by the following formula:

(*2*) $$\text{VIF}=\frac{Var(E)}{Var(X)},$$

where *Var*(*E*) reflects the mean estimation variance of all regression weights (stimulation and information storage regressors for each finger) whereas *Var*(*X*) reflects the mean estimation variance in case all regressors would be estimated individually. A VIF of 1 indicates a total absence of collinearity between the regressor of interest and all other regressors in our GLM whereas a large VIF signals a serious collinearity problem. There is no clear threshold for acceptable multicollinearity. Previous literature however recommends that the VIF is ideally smaller than 2.5 (55). In our case, the VIF was 1.45, averaging across regressors reflecting the first stimulation, the delay, and the second stimulation.

### Multivariate Pattern Analysis

#### MVPA.

We used multivoxel pattern analysis (MVPA) to decode which finger was stimulated based on activity during the delay period. This analysis was conducted for voxels within the S1 hand area mask that have been shown to possess fine-grained finger representations. First-level parameter estimates were computed for all events of each trial and each participant using a voxel-based general linear model (GLM) in SPM (v.12) based on the γ hemodynamic response function. This resulted in 192 β estimates (48 trials × 4 runs) during the delay period per participant across both conditions. Ninety-six β estimates for each memory and no-memory condition.

We trained a linear classifier (support vector machine, SVM) to predict which finger was stimulated in a specific trail based on the respective delay period activity using the nilearn toolbox (56). We calculated classification accuracies using a leave-one-run-out cross-validation approach. The accuracies were averaged across folds, resulting in one accuracy per condition and per participant.

#### Representational similarity analysis.

Representational similarity analysis (RSA) has the ability to identify the invariant representational structure of fingers independent of amplitude, shape, and exact location of activated brain regions during the WM task (36). It allowed us to obtain a measure of how distinguishable somatotopic representations between working-memory and no-working-memory trials are. We computed representational distances between activity patterns related to different fingers (index vs. little finger) for both working-memory and no-working-memory conditions. The distances were obtained using a prewhitened crossvalidated Mahalanobis distances [crossnobis distances (51, 57)]. We obtained voxel-wise parameter estimates (βs) for each finger × memory condition × time point versus rest (using univariate analysis) and residuals of our GLM within the hand area of S1. These βs were prewhitened using the residuals. Based on the prewhitened βs, we computed squared Mahalanobis distances between all possible finger × condition × time point combinations for each fold (i.e., run) and averaged them across folds. A distance greater than 0 reflects dissociable cortical representations whereas 0 shows no dissociation. The distance measures between all possible representations were assembled in a representational dissimilarity matrix (RDM). For visualization, we only extracted distances between the finger representations during memory versus no-memory for each time point (f1, delay, and f2) of the task.

### Statistical Data Analysis

To detect outliers, we used the robustbase toolbox (58). *S_n_* identifies an outlier (*x_i_*) if the median distance of *x_i_* from all other points was greater than the outlier criterion (λ = 3) times the median absolute distance of every point from every other point:

(*3*) $$\;\frac{{\text{med}}_{j\neq i}\;|\;x_{i}-x_{j\;}|}{S_{n}}>\lambda \text{ where }S_{n}=\;{c_{n}}_{i=1:n}^{\mathrm{med}}\{\begin{array}{c} \mathrm{med}\\ j\neq 1 \end{array}|x_{i}-x_{j}\},$$

where *c_n_* is a bias correction factor for finite sample sizes (59). We detected no outliers for behavioral data that had to be excluded from any further analysis.

Before conducting any repeated-measures ANOVA testing, we validated the assumptions for normality and sphericity using a Shapiro–Wilk and Mauchy test. Effect sizes of different variables were measured using η^2^. ANOVA analysis was done using the pingouin toolbox (60). *P* values were Greenhouse–Geisser corrected if sphericity could not be assumed. *T* statistics were corrected for multiple comparisons using one-step Bonferroni correction.

Bayesian analysis was carried out using pingouin toolbox for the main comparisons to investigate support for the null hypothesis. Following the conventional cut-offs, a Bayes factor (BF) smaller than 1/3 is considered substantial evidence in favor of the null hypothesis. A BF greater than 3 is considered substantial evidence, and a BF greater than 10 is considered strong evidence in favor of the alternative hypothesis. A BF between 1/3 and 3 is considered weak or anecdotal evidence (61, 62).

## RESULTS

### Behavioral Performance Was Better with Greater Frequency Differences

**Figure 2.**
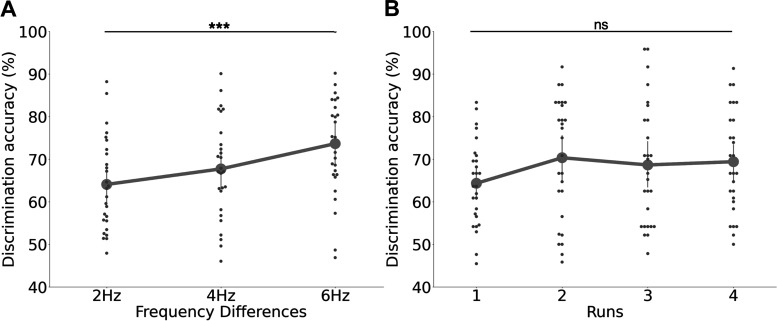
Behavioral performance results. *A*: discrimination accuracy (% of correct answers) improved when frequency differences were larger. The thick gray dots reflect the group mean and the error bars indicate the standard deviation. Small gray dots represent individual participants’ results. *B*: behavioral performance was not significantly different across runs. This demonstrates that our sensory detection threshold (SDT) criterion assured stable discrimination accuracies across runs. ***P < 0.001; ns, nonsignificant.

A two-way ANOVA showed that behavioral performances differed significantly depending on the frequency differences between the first and second vibrotactile stimulus [*F*(2,156) = 5.06, *P* < 0.001, η^2^ = 0.06; Fig. 2*A*], but not between stimulated fingers [*F*(1,156) = 0.44, *P* = 0.51, η^2^ < 0.01]. There was no interaction effect [*F*(1,156) = 0.88, *P* = 0.42, η^2^ = 0.01]. A post hoc test [Tukey’s honest significant difference (HSD)] on pairwise comparisons on frequency differences pairs revealed that discrimination accuracy was significantly different between 2 and 6 Hz differences (*q =* 4.48, *P* < 0.01) and showed no significant difference for the rest of the frequency difference pairs (4 Hz vs. 6 Hz: *q =* 2.67, *P* = 0.15 and 2 Hz vs. 4 Hz *q =* 1.81, *P* = 0.41). We further analyzed whether behavioral performance changed across runs despite our efforts to re-adjust the detection threshold (Fig. 2*B*) did not find significant differences across runs [*F*(3,2496) = 2.0, *P* = 0.11, η^2^ <0.01] or across fingers [*F*(1,2496) = 0.48, *P* = 0.49, η^2^ < 0.01], and no significant interaction effect [*F*(3,2496) = 0.47, *P* = 0.47, η^2^ < 0.01].

### Vibrotactile Working Memory Recruits a Distributed Brain Network

**Figure 3.**
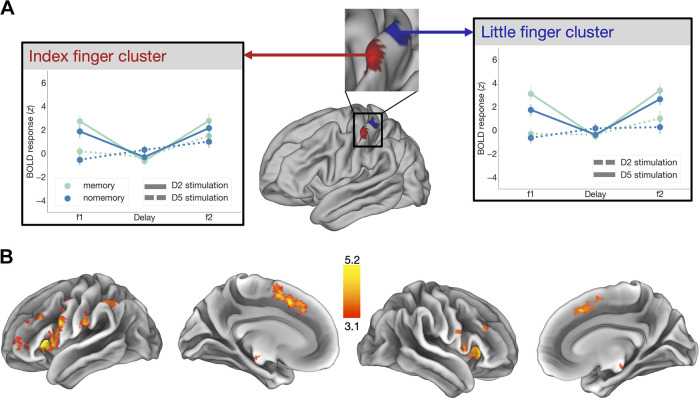
Univariate group results. *A*: S1 areas activated during index (red) and little (blue) stimulations (f1 and f2 combined, *middle*). Within each participant’s individual local maxima sphere (*r* = 6 mm) closest to the group finger peak within each cluster (index finger = *upper left* and little finger = *upper right*), blood oxygen level-dependent (BOLD) responses to either corresponding (highlighted) or not corresponding (lower opacity) finger stimulations are displayed as group averages across different time points (f1, delay, and f2). Point plots are centered at the mean and error bars reflect the standard error. *B*: we determined brain regions that were more activated during the delay period of the memory compared to the no-memory condition. A statistical map (*Z* > 3.1) was obtained by contrasting delay period activity in memory trials to no-memory trials. The map was separately projected onto a cortical surface contralateral (*top*) and ipsilateral (*bottom*) to the stimulus site. Activity related to actively keeping the vibrotactile task in memory resided in a wider network of brain regions (for more details, see Table 1).

We contrasted activity levels during vibrotactile stimulation between fingers (i.e., index > little and little > index finger), and, as expected, observed separated finger representations in contralateral S1 (Fig. 3*A*, *middle*) with the little finger being represented more medially than the index finger (6–9, 11, 35). These representations greatly overlapped during both vibrotactile stimulation timepoints (f1 and f2, Supplemental Fig. S4, https://doi.org/10.6084/m9.figshare.23947041). Finally, we assessed whether finger-specific activity (e.g., index finger-related activity which was greater within the index finger cluster) was modulated within these finger-specific clusters during memory compared with no-memory trials at different timepoints of the task (f1, delay, and f2). Our results revealed that the memory condition led to stronger BOLD responses during vibrotactile stimulations (f1 and f2), but not during the delay period for both index finger [timepoints main effect: *F*(2,50) = 66.48, *P*_corr_ < 0.001, η^2^ = 0.64; conditions main effect: *F*(1, 25) = 12.74, *P*_corr_ < 0.01, η^2^ < 0.05; interaction effect: *F*(2,50) = 12.15, *P*_corr_ < 0.001, η^2^ < 0.05; pairwise comparisons: f1 memory versus f1 no-memory: *t*(25) = 3.93, *P*_corr_ < 0.01; BF10 = 54.8, delay memory vs. delay no-memory: *t*(25) = −2.3, *P*_corr_ = 0.09; BF10 = 1.903, f2 memory vs. f2 no-memory: *t*(25) = 3.49, *P*_corr_ < 0.01; BF10 = 20.57, Fig. 3*A*, *left*] and little finger cluster [time point main effect: *F*(2,50) = 73.27, *P*_corr_ < 0.001, η^2^ = 0.66; condition main effect: *F*(1,25) = 41.63, *P*_corr_ < 0.001, η^2^ < 0.05; interaction effect: *F*(2,50) = 15.43, *P*_corr_ < 0.001, η^2^ < .05; pairwise comparisons: f1 memory vs. f1 no-memory: *t*(25) = 8.05, *P*_corr_ < .001; BF10 > 100, delay memory vs. delay no-memory: *t*(25) = −2.3, *P*_corr_ = 0.51; BF10 = 0.25, f2 memory vs. f2 no-memory: *t*(25) = 3.93, *P*_corr_ < 0.01; BF10 = 54.4, Fig. 3*A*, *right*].

We also determined brain areas that were more activated during the delay period in the memory compared with the no-memory condition. We found that actively retaining vibrotactile information involved a distributed brain network: i.e., bilateral frontal lobe, bilateral (medial/inferior) frontal gyrus (MFG, IFG), bilateral premotor cortex, and supplementary motor area (PMC, SMA), contralateral secondary somatosensory cortex (S2), bilateral inferior parietal lobule (IPL), bilateral superior parietal lobule (SPL), bilateral supramarginal gyrus (SMG), bilateral caudate, bilateral thalamus, bilateral nucleus accumbens, and bilateral insula (Fig. 3*B*, Table 1 and Supplemental Fig. S1, https://doi.org/10.6084/m9.figshare.23946948). This network has been previously identified in tactile WM tasks and, as in previous human fMRI studies, this univariate analysis did not reveal significant S1 activity.

**Table 1.** Brain regions that were significantly activated by keeping the tactile task in memory during the delay period as shown in Fig. 3B (memory > no-memory trails)

Anatomical Region	Peak MNI Coordinates			Mean Fisher *Z*	V-Shaped Activity
	*X*	*Y*	*Z*		
Left frontal pole	−42	52	2	4.33	3.29
Right frontal pole	40	46	32	3.86	3.16
Left IFG	−52	16	2	5.14	6.44
Right IFG	58	10	18	4.59	5.47
Left MFG	−44	30	34	4.45	
Left PMC (+SMA)	−4	8	50	5.49	5.81
Right PMC (+SMA)	8	20	46	5.78	5.66
Left S2	−56	−23	20	3.25	5.75
Right S2	54	−14	16		5.14
Left S1	−54	−22	46		5.63
Right S1	56	−16	40		5.75
Left IPS	−42	−52	48	5.14	
Left IPL	−56	−20	28	4.78	5.52
Right IPL	46	−48	52	4.86	5.67
Left SMG	−42	−46	40	4.77	
Right SMG	51	−32	46	4.16	
Left SPL	−50	−48	58	4.64	4.97
Left insular cortex	−30	24	6	5.61	5.41
Right insular cortex	36	22	−2	5.41	5.32
Left accumbens	−12	14	−6	5.27	
Right accumbens	10	12	−4	4.16	
Left putamen	−18	6	−10	4.96	4.42
Right putamen	20	10	−8	4.34	3.71
Left caudate	−12	18	0	4.80	5.45
Right caudate	14	18	4	4.97	4.32
Left thalamus	−8	−24	−16	3.86	3.81
Right thalamus	2	−20	−12	3.91	3.99

In the next step, we parametrically modulated the delay activity, assuming a V-shaped activity during the delay period. These *z-*stats results were added as an additional column to the table (very right). All *z*-statistic images were thresholded using clusters determined by *Z* > 3.1 and *P* < 0.05 family-wise-error-corrected (FWE) cluster significance. Mean Fischer *Z* indicates peak *z*-values. Areas were labeled according to the Juelich Histological Atlas and Harvard-Oxford (Sub-) Cortical Structural Atlas (63). IFG, inferior frontal gyrus; IPL, inferior parietal lobule; IPS, intraparietal sulcus; MFG, medial frontal gyrus; MNI, Montreal Neurological Institute; PMC, premotor cortex; S1, primary somatosensory cortex; S2, secondary somatosensory cortex; SMA, supplementary motor area; SMG, supramarginal gyrus; SPL, superior parietal lobule.

### Temporal Modulation of Delay Period Activity in Contralateral S1

**Figure 4.**
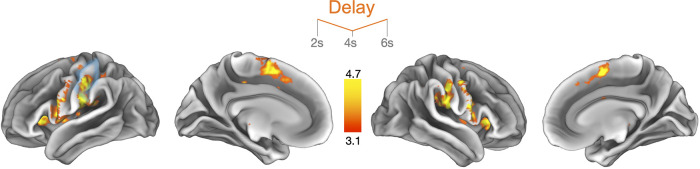
V-shaped parametric modulation of brain activity during the delay period. Brain regions exhibiting V-shaped modulated delay activity patterns (see *inset* at the top reflecting the parametric modulator entered into the general linear model, GLM) during the delay period (2–6 s; for more details, see Table 1). The contrast shows the difference between the memory and no-memory condition in the contralateral (*left*) and ipsilateral hemisphere (*right*). The area highlighted in blue represents the primary somatosensory cortex (S1) hand area.

We were interested in examining whether brain activity temporally changed during the delay period. We parametrically modulated the delay period regressors by the hypothesized V-shaped activity changes and computed the associated statistical maps. A V-shaped activity modulation was found in a similar network of brain regions as for the aforementioned univariate results (Fig. 4). In addition, we also found significant changes in BOLD signal in bilateral S1 and S2. S1 activity overlapped with the area that usually represents the hand. Detailed differences in results between both analysis approaches are displayed in Table 1.

For visualization purposes only, we extracted the activity at each time bin (i.e., from a separate GLM not involving a parametric modulation regressor) within the contralateral S1 hand area to demonstrate V-shaped activity across time bins (Supplemental Fig. S3, https://doi.org/10.6084/m9.figshare.23946978).

### Finger-Specific S1 Representations Are Modulated by Performing the Vibrotactile Discrimination Task

**Figure 5.**
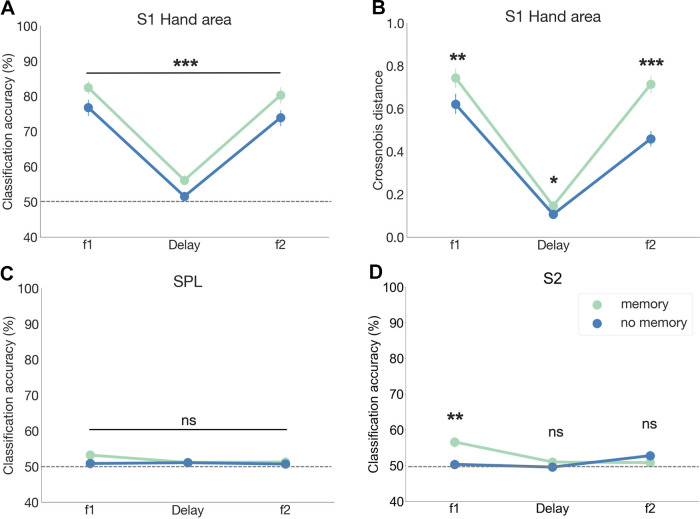
Multivariate results on somatotopic modulations. We investigated whether activity patterns within a region of interest (ROI) with fine-grained finger somatotopy became more distinct at different time points (f1, delay, and f2) during memory trials compared with no-memory trials. *A*: classification accuracies (index vs. little finger) based on activity patterns in hand area within the contralateral primary somatosensory cortex (S1). *B*: cross-validated Mahalanobis (crossnobis) distances between fingers × condition in the same ROI. *C* and *D:* further explorative analysis of ROIs that previously were indicated to be involved in vibrotactile working memory (WM), but where no fine-grained somatotopy is assumed. The point plots are centered at the mean and error bars reflect the standard error. Gray dotted lines reflect the theoretical chance level. If interaction effects were significant, pairwise comparison results for comparing memory vs. no-memory conditions separately for each time point are indicated by *P < 0.05, **P < 0. 01, ***P < 0.001. S1, primary somatosensory cortex; S2, secondary somatosensory cortex; SPL, superior parietal lobule.

We hypothesized that performing the vibrotactile discrimination task would modulate finger-specific representations in S1. To test this hypothesis, we used MVPA to decode the stimulated finger (i.e., index vs. little finger) during the first stimulation (f1), during the delay period, and the second stimulation (f2) separately for memory and no-memory trials (Fig. 5*A*). We did this in the contralateral S1 ROI, which has shown to possess fine-grained finger representations (5–9). Our results revealed that we were able to decode from the activity patterns in contralateral S1 with greater classification accuracies which finger was stimulated during memory trials compared with no-memory trials [condition main effect: *F*(1,26) = 48.42, *P*_corr_ < 0.001, η^2^ < 0.05]. Classifications accuracies differed across different time points [f1, delay, and f2; time point main effect: *F*(2,52) = 156.52, *P*_corr_ < 0.001, η^2^ = 0.77]. However, we found no time × condition interaction effect [*F*(2,52) = 0.5, *P*_corr_ = 0.59, η^2^ < 0.001].

Furthermore, finger-specific representations in S1 can also be investigated in multivoxel information space and the patterns of activation in that space could be referred to as the “representational geometry” of memorized tactile stimuli (Fig. 5*B*). The distance in geometrical space provides a straightforward measure to determine whether stimulating the index finger versus the little finger elicits distinct or partially overlapping patterns of brain activity in S1. Our repeated-measures ANOVA revealed that the cross-validated Mahalanobis (crossnobis) distances obtained from contralateral S1 hand area were significantly higher during the stimulation periods (f, f2) than during the delay period [time point main effect: *F*(2,52) = 151.21, *P*_corr_ < 0.001, η^2^ = 0.73] and during the memory compared with the no-memory trials [condition main effect: *F*(1,26) = 39.81, *P*_corr_ < 0.001, η^2^ = 0.06]. We also found an interaction effect [i.e., *F*(2,52) = 28.65, *P*_corr_ < 0.001, η^2^ < 0.05] indicating that the task-specific modulation of the representational distance was larger during the stimulation periods than the delay period. We again could show that the geometrical distance of finger representations, as measured by crossnobis distances, between memory versus no-memory trials reached significance when independently tested for vibrotactile stimulations [f1: *t*(26) = 4.09, *P*_corr_ < 0.01; BF10 = 81.73; f2: *t*(26) = 7.4, *P*_corr_ < 0.001; BF10 > 100] and also for the delay period [i.e., in the absence of any tactile stimulation; *t*(26) = 2.9, *P*_corr_ < 0.05; BF10 = 5.96, with the Bayes factor (BF) showing substantial evidence in favor of the null hypothesis].

We also explored further ROIs located on the superior parietal lobe (SPL; Fig. 5*C*), inferior frontal gyrus (IFG), and secondary somatosensory cortex (S2; Fig. 5*D*) that have been implicated to be involved in vibrotactile WM (23, 29, 31). ROIs were based on clusters obtained in univariate analysis (Delay_Memory_ > Delay_noMemory_). We found no significant differences in classification accuracies based on activity patterns within contralateral SPL [time point main effect, *F*(2,52) = 0.75, *P*_corr_ = 0.46, η^2^ < 0.05; condition main effect, *F*(1,26) = 0.85, *P*_corr_ = 0.37, η^2^ < 0.05; and time point × condition interaction effect, *F*(2,52) = 0.68, *P*_corr_ = 0.51, η^2^ < 0.05) or IFG (time point main effect, *F*(2,52) = 0.93, *P*_corr_ = 0.4, η^2^ < 0.05; condition main effect, *F*(1,26) = 0.38, *P*_corr_ = 0.55, η^2^ < 0.05; and time point × condition interaction effect, *F*(2,52) = 1.28, *P*_corr_ = 0.28, η^2^ < 0.05]. Within contralateral S2, our results revealed that only during the first stimulation (i.e., during stimulus encoding), classification accuracies significantly differed between conditions, but not for the delay nor second stimulation [time point main effect, *F*(2,52) = 3.63, *P*_corr_ < 0.05, η^2^ < 0.05; condition main effect, *F*(1,26) = 4.56, *P*_corr_ < 0.05, η^2^ < 0.05 and time point × condition interaction effect, *F*(2,52) = 6.13, *P*_corr_ < .01, η^2^ < 0.05; pairwise comparisons: f1 memory versus f1 no-memory: *t*(26) = 3.46, *P*_corr_ < 0.01; BF10 = 19.72, delay memory versus delay no-memory: *t*(26) = 1.33, *P*_corr_ = 0.59; BF10 = 0.45, f2 memory versus f2 no-memory: *t*(26) = −1.05, *P*_corr_ = 0.91; BF10 = 0.33]. Our results suggest that during the delay period of a vibrotactile WM task, modulation of finger representations is only observed in S1 and not in other areas of the WM network (i.e., SPL, and S2).

## DISCUSSION

In the present study, we demonstrated by using MVPA that the representation of finger-specific, somatosensory information in S1 is modulated by cognitive processes underlying a vibrotactile discrimination task. This was the case during the tactile stimulation periods but also during the delay period (i.e., in the absence of any tactile stimulation). We propose that cognitive demands significantly modulated finger representations in S1, probably due to top-down control mechanisms that might be associated with attentional control or memory processes which, in turn, sharpen tuning curves of neurons in S1.

### Modulation of Finger Representations during Stimulus Perception

Our participants were able to perform the vibrotactile discrimination task inside the scanner and exhibited the expected effect that discrimination accuracy (% of correct answers) improved when the frequency differences between the stimuli were larger. It has to be noted that intensity cues are an important sensory attribute of vibrotactile stimulation that can provide useful information about the strength of the tactile input (64, 65). Even though the stimulation frequency varied within a rather small range (20–26 Hz), it is possible that these frequency differences have caused small changes in the perceived intensity of the stimulation (66) that might have contributed to performing the discrimination task. Note that we did not try to decode stimulus characteristics per se from brain activity but rather investigated how solving this task influences somatotopic representations of the index versus ring finger. In summary, our behavioral data confirm that participants discriminated between stimuli and that cognitive demands differed between the memory and no-memory trials.

When exposed to a stimulus, stimulus-selective neurons are activated that can be quantified using tuning curves. Previously, specific neurons have shown “tuned” responses to different features of the stimulus, e.g., stimulus location or stimulus orientation (67–69). For instance, responses of neuronal populations in the primary visual cortex (V1) can be modulated by changes in stimulus orientation (70). Similarly, neural tuning also determines whether different stimuli evoke rather distinct or similar response patterns in the multivoxel space of S1, which can be quantified by RSA as a distance within the representational geometry (28). A larger distance indicates that stimuli applied to the index versus the little finger evoke more distinct activity patterns in S1. Our data indicates that performing a vibrotactile discrimination task either with the index or the little finger caused representations to be more distinct (i.e., larger distance within the representational geometry) consistent with a narrower neural tuning within S1. Interestingly, this task effect was not only present during stimulus presentation but, to an extent, also during the delay period (Fig. 5*B*).

It is uncertain which specific cognitive mechanism might have driven the observed S1 modulation. A discrimination task usually requires enhancing neural activity related to relevant stimuli and suppressing activity related to irrelevant stimuli. Such amplification of relevant information has been conceptualized as generalized models of attention (for a review, see Ref. 71). Attention has been suggested as an integral part of performing tasks with a WM component (14, 31, 72, 73) as it filters information by sensory modality or by body location (74) and, thereby, attention regulates what will be cortically represented and what will not (75). Volitionally directing attention toward a spot on the body surface that was tactilely stimulated (15, 76, 77) or expected to be stimulated (78) increases BOLD responses in a somatotopic manner within S1. Our multivariate results support this notion. During both time points of stimulation (f1 and f2), we obtained higher classification accuracies and crossnobis distances for the memory than the no-memory condition from activity patterns in the S1 hand area. This finding is in line with the accumulating evidence that attention modulates tuning curves in the specific sensory modality (79–81). In our case, shifting attention to either the index or little finger would relocate the spotlight of the attentional field accordingly to modulate responses in those voxels that somatotopically represent the attended finger.

### Modulation of Finger Representations during the Delay Period

Our analysis of the delay period revealed a widespread parieto-fronto-insular network that is typically involved in tactile WM (22, 24). We could show that fronto-partial areas, insular cortices, subcortical regions (i.e., caudate and thalamus), S1, and S2 demonstrated a V-shaped activity profile across the delay period, which might provide a glimpse into the temporal modulation of WM-related brain activity (82). Task-relevant modulation could occur through the prefrontal cortex (PFC) by modulating cortical representations of WM contents via top-down control to guide behavior (for review, see Ref. 83).

Moreover, the MVPA analyses revealed that finger representations in S1 but not in S2 or other WM-related areas were modulated by keeping a tactile stimulus in memory, even though it is unclear which specific mechanism has driven this effect. The general involvement of S1 is in line with previous neuroimaging studies in humans suggesting that tactospatial information is retained during the delay period by a neural population code in S1, but also in SPL, PMC, and posterior parietal cortex (29, 31). We were unable to find any evidence of S2 involvement, which had previously been suggested (23). This could be attributed to less somatotopy in this region, which may not be discernible by the coarse spatial resolution of our fMRI measurements (84).

However, it is important to note that we did not decode stimulus characteristics per se but rather which finger performed the vibrotactile discrimination task. Therefore, it seems more likely that our results reflect an attentional mechanism. Models of attention-based rehearsal assume that spatial attention contributes to spatial contents of WM (85–87). This could occur through focusing attention to memorized locations during the delay period of the WM tasks (88, 89) which can enhance memory accuracy (86, 87, 90). Thus, we tentatively suggest that greater representational dissimilarities during the delay period might reflect attentional top-down control that has tuned somatotopic finger representation optimally for performing the vibrotactile discrimination task, a phenomenon that might still be detectable even in the absence of tactile input. However, this interpretation requires further confirmation since the memory delay period in our experiment was relatively short (6–8 s) compared with other fMRI studies investigating WM that often used longer delays or masking to reduce the influence of carry-over effects of the first tactile stimulus (30, 31). The low VIF of the regressors suggests that activity related to stimulus perception and WM storage could be disentangled by our model but future studies might use better-tailored paradigms to dissociate whether finger representations in S1 are modulated mainly by representing tactile stimuli in memory or by directing attention to the task-relevant effector.

### Conclusions

Our results extend previous findings on somatotopic representations of S1 by confirming that solving a vibrotactile discrimination task modulates finger representations. Higher classification accuracies and larger dissimilarities between finger representations suggest that neural tuning was sharpened, most likely due to top-down attentional mechanisms.

## DATA AVAILABILITY

Source data for this study are not publicly available due to privacy or ethical restrictions. The source data are available to verified researchers upon request by contacting the corresponding author.

## SUPPLEMENTAL DATA

10.6084/m9.figshare.23946948 Supplemental Fig. S1: https://doi.org/10.6084/m9.figshare.23946948.

10.6084/m9.figshare.23946972 Supplemental Fig. S2: https://doi.org/10.6084/m9.figshare.23946972.

10.6084/m9.figshare.23946978 Supplemental Fig. S3: https://doi.org/10.6084/m9.figshare.23946978.

10.6084/m9.figshare.23947041 Supplemental Fig. S4: https://doi.org/10.6084/m9.figshare.23947041.

10.6084/m9.figshare.24049257 Supplemental Table S5: https://doi.org/10.6084/m9.figshare.24049257.

## GRANTS

This work was supported by the Swiss National Science Foundation Grants 320030_175616 and 32003B_207719 and by the National Research Foundation, Prime Minister’s Office, Singapore under its Campus for Research Excellence and Technological Enterprise (CREATE) program (FHT). S.K. was supported by the Swiss National Science Foundation Ambizione Grant PZ00P3_208996.

## DISCLOSURES

No conflicts of interest, financial or otherwise, are declared by the authors.

## AUTHOR CONTRIBUTIONS

F.R., S.K., and N.W. conceived and designed research; F.R. performed experiments; F.R. analyzed data; F.R., S.K., and N.W. interpreted results of experiments; F.R. prepared figures; F.R. drafted manuscript; F.R., S.K., and N.W. edited and revised manuscript; F.R., S.K., and N.W. approved final version of manuscript.
